# Hepatitis A Virus Incidence Rates and Biomarker Dynamics for Plasma Donors, United States

**DOI:** 10.3201/eid2711.204642

**Published:** 2021-11

**Authors:** Stephanie Schoch, Martin Wälti, Mathias Schemmerer, Rick Alexander, Björn Keiner, Carol Kralicek, Keith Bycholski, Kelley Hyatt, Jon Knowles, Denis Klochkov, Toby Simon, Jürgen J. Wenzel, Nathan J. Roth, Eleonora Widmer

**Affiliations:** CSL Behring AG, Bern, Switzerland (S. Schoch, M. Wälti, D. Klochkov, N.J. Roth, E. Widmer);; University Medical Center Regensburg, Regensburg, Germany (M. Schemmerer, J.J. Wenzel);; CSL Plasma, Knoxville, Tennessee, USA (R. Alexander);; CSL Behring GmbH, Marburg, Germany (B. Keiner);; CSL Plasma, Boca Raton, Florida, USA (K. Bycholski, K. Hyatt, J. Knowles, T. Simon);; CSL Plasma, Dallas, Texas, USA (C. Kralicek)

**Keywords:** hepatitis A, hepatitis A virus, HAV, viruses, hepatitis, RNA, IgM, IgG, alanine aminotransferase, ALT, virus doubling time, RNAemia, clinical markers, outbreak, incidence rates, biomarker dynamics, plasma donors, genotypes, United States

## Abstract

The United States is currently affected by widespread hepatitis A virus (HAV) outbreaks. We investigated HAV incidence rates among source plasma donors in the United States since 2016. Serial donations from HAV-positive frequent donors were analyzed for common biologic markers to obtain a detailed picture of the course of infection. We found a considerable increase in incidence rates with shifting outbreak hotspots over time. Although individual biomarker profiles were highly variable, HAV RNA typically had a high peak and a biphasic decrease and often remained detectable for several months. One donor had a biomarker pattern indicative of previous exposure. Our findings show that current HAV outbreaks have been spilling over into the plasma donor population. The detailed results presented improve our comprehension of HAV infection and related public health aspects. In addition, the capture of full RNA curves enables estimation of HAV doubling time.

Hepatitis A virus (HAV) is a small, nonenveloped RNA virus belonging to the family *Picornaviridae*. Its biology and transmission cycle have been reviewed elsewhere (*1*,*2*) and are therefore only briefly introduced. Six genotypes and 1 serotype have been described; genotypes I‒III circulating in humans and IV‒VI circulating in nonhuman primates. Virions exist in either of 2 forms (*3*): lipid-associated (main form in blood, also referred to as quasi-enveloped) or truly nonenveloped (main form in stool). Although parenteral transmission has also been reported, the predominant transmission route is the fecal‒oral through contact with infected persons or uptake of contaminated food and water. Infection might involve an early, as of yet poorly characterized, extrahepatic replication phase (e.g., in gut epithelial cells [*4*]); from the gut, virions are then transported through the blood to their primary replication site, the liver. The transmission cycle ends with a transport of viral progeny via the bile to the gut, leading to massive virus shedding in stool (*2*). Although the course of disease is generally self-limiting, several serious complications can occur, especially in older persons or in combination with risk factors.

Since 1995, when the first HAV vaccine was licensed in the United States, the annual US incidence rate of acute hepatitis A has decreased tremendously (*1*,*5*). During 2015, the National Notifiable Diseases Surveillance System of the Centers for Disease Control and Prevention (CDC) recorded an annual average of 0.4 cases/100,000 inhabitants (*5*). However, since 2016, the downward trend has reversed (*6*). In mid-2016‒early 2017, Michigan, California, Kentucky, and Utah began to report local person-to-person HAV outbreaks, which have since become a national concern: 38,031 cases affecting 35 states (status as of February 2021) (*7*). In 2018, the annual US incidence rate was 3.8 cases/100,000 population, and the true rate was estimated to be twice as high because of under-ascertainment and under-reporting (*6*). The current outbreak is attributable to the fact that most (≈74%) of US-born adults are susceptible to HAV (*8*). This pattern is typical for industrialized countries that have good standards of sanitation and hygiene and a history of restrictive (mostly infant-targeted or risk group‒targeted) vaccination practices (*9*). HAV genotype IB has been the most common genotype during this outbreak, whereas before 2017, most cases in the United States involved genotype IA (*10*).

Human plasma is used as a starting material to produce several life-saving therapies, such as immunoglobulins. The safety of these products with regard to transmission of bloodborne viruses is based on 3 pillars: selecting low-risk donors, testing for relevant viral markers, and including process steps capable of removing or inactivating a broad range of viruses.

To ensure a reliable supply of therapies, CSL operates a large collection center network for source plasma, which is plasma serially collected from healthy, voluntary donors through plasmapheresis. CSL Plasma, a division of CSL, operates one of the largest global plasma collection networks, consisting of >260 collection centers throughout the United States. Each donation collected is tested for HAV RNA. These data provide a unique glimpse into how the HAV epidemiology trends in the United States have changed. Unlike whole blood donors, plasma donors are allowed to donate frequently, which can be useful from a research perspective.

During 2017, we noticed a trend toward higher HAV incidence rates among US donors. Because knowledge on the course of infection and host response during asymptomatic/subclinical infection is scant, we conducted this study. The aim of this study was to assess the effects of the current HAV outbreak on plasma donors in the United Sates and to complement existing knowledge on HAV biomarker dynamics.

## Materials and Methods

### Plasma and Routine Viral Marker Testing

Source plasma donors in the United States can donate <2 donations/week. Plasma donations and matching samples for routine viral marker testing (HIV, hepatitis B virus [HBV], hepatitis C virus [HCV], HAV, and parvovirus B19) were collected from donors by plasmapheresis in 4% sodium citrate and frozen at <‒30°C. The freezing process was compliant with the European Pharmacopeia (*11*). We performed routine, qualitative nucleic acid testing (NAT) for HAV by using the Roche Cobas DPX Test (https://diagnostics.roche.com) on minipools of <96 donations. In the format used, the 95% limit of detection (LOD) of the assay was ≈105.6 IU/mL of HAV RNA/individual donation. We subjected HAV-positive minipools to resolution testing down to the individual donation. For all donors with a first HAV-positive donation, all previous/subsequent donations from a defined time period were put on quarantine and excluded from use for fractionation.

### Donor Selection

We retrospectively included in this study 10 qualified US-source plasma donors with ≥1 HAV-positive NAT result; these donors had met all medical criteria for donation (*12*). To ensure optimal coverage of the viremic phase, donors had to fulfill 3 additional criteria: first, >10 serial donations from 1 month before to 2 months after the first HAV-positive donation available; second, >1 nonreactive donation preceding the first positive donation; and third, some coverage of the late phase of infection. The aim was to analyze all donations collected during the period −30 to +120 days of the first HAV NAT-positive donation (day 0). Nevertheless, for 7 of 10 donors, 1‒7 donations were unavailable (plasma discarded or used for other research purposes).

### Nonroutine Biomarker Testing

Testing was performed by accredited contract-testing laboratories using validated assays after having received approval for the study protocol by the WIRB Copernicus Group Institutional Review Board (https://www.wcgirb.com). Samples used were deidentified aliquots of quarantined donations stored at <‒20°C; they had been subjected to 2 freeze‒thaw cycles at the time of testing. We tested the following biomarkers at the individual donation level for each sample: HAV RNA, liver injury marker alanine aminotransferase (ALT), and HAV IgM and IgG.

HAV RNA was quantified at Interregional Blood Transfusion, Swiss Red Cross (IRB SRC), Bern, Switzerland (https://www.redcross.ch), by using a NAT assay targeted against the HAV 5′-noncoding region, which had a validated 95% LOD of 14 IU/mL and a linear range of 81.6‒1.1 × 10^8^ IU/mL (*13*). The remaining analytics were performed at the National Reference Laboratory for HAV, University Medical Center Regensburg, Regensburg, Germany. ALT was measured by using the quantitative Roche Cobas ALT Assay (Roche reference no. 05850797–190), which had a validated LOD of 5 IU/L for serum and upper limits of reference ranges of 35 IU/L for women and 50 IU/L for men. A control experiment with ALT-spiked samples confirmed that the citrate plasma matrix had no major impact on readout of the assay: at 50 IU/L and 150 IU/L ALT, average readouts for spiked serum and citrate plasma samples were within <5% of each other. A total of 13 ALT results from 3 donors were invalid because samples exceeded the lipemia threshold. We analyzed HAV IgM and IgG by using the Abbott Architect HAVAb IgM and IgG Assays (https://www.abbott.com). Finally, we identified the HAV genotype by sequencing HAV coat protein viral protein 1/core protein P2A regions as described (*14*).

### HAV Doubling Time

We calculated doubling time (Td) by using the formula Td = ln(2)/B based on an exponential trendline (y = Ae^Bx^) determined in Excel (Microsoft, https://www.microsoft.com) for each donor’s HAV RNA growth curve. In these equations A and B represent calculated coefficients standing for HAV RNA initial amount (A) and growth rate (B), e is base of the natural logarithm, x is time in days, and y is HAV RNA titer in IU/mL To focus exclusively on the logarithmic growth phase, we excluded samples with a positive HAV IgM or IgG result and from visibly flattened areas of the RNA curve. Donor E was excluded from the analysis because the early growth phase was not represented.

## Results

### HAV Incidence Rates for Plasma Donors 

**Figure 1:**
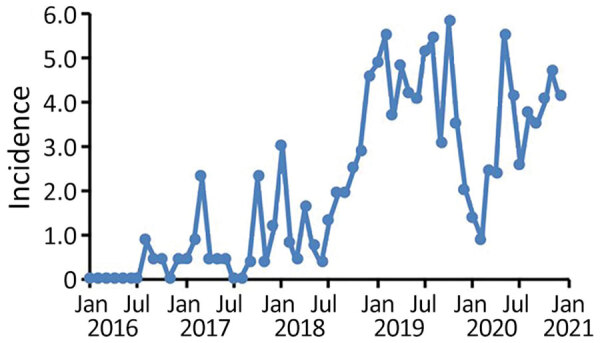
Incidence rates for hepatitis A virus (HAV) in source plasma donors, United States, 2016‒2020. Shown are monthly HAV incidence rates based on routine viral marker testing. Incidence rates correspond to monthly number of previously HAV RNA-negative donors with a first positive HAV nucleic acid testing result per 100,000 donors.

During January 2016‒December 2020, a total of 348 different donors from the United States donated plasma that tested positive for HAV RNA; these donations were excluded from further manufacturing processes. Monthly HAV incidence rates derived from these data showed a considerable increase from typically 0.0 cases/100,000 donors at the beginning of 2016 to a peak rate of 5.8 cases/100,000 donors during October 2019 (Figure 1). The highest rates were found during November 2018‒November 2019; most cases were found in the states of Indiana, Ohio, Tennessee, Pennsylvania, and Florida.

**Figure 2:**
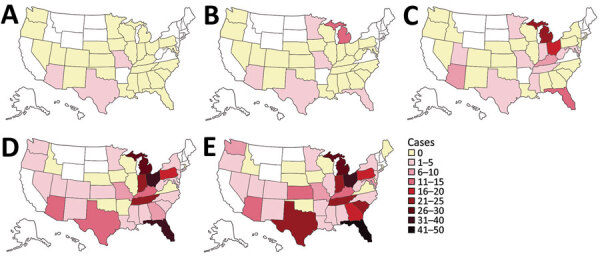
Geographic distribution of hepatitis A virus–positive plasma donors, United States, 2016‒2020. Cumulative case counts are indicated: A) 2016; B) 2016–2017; C) 2016–2018; D) 2016–2019; E) 2016–2020. These counts reflect how hepatitis A outbreaks in the United States have spread and spilled over into the plasma donor population over time. No color indicates states for which no data are available (no plasma collection). Maps were created by using an Adobe Stock template (https://stock.adobe.com).

**Figure 3:**
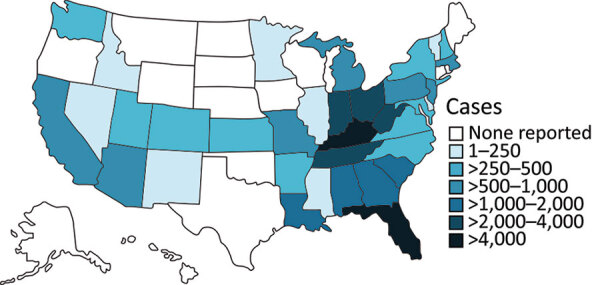
Geographic distribution of CDC-reported HAV outbreak cases, United States, as of February 2021, showing state-reported HAV outbreak cases as listed on the CDC website for the current outbreak since August 2016 (*7*). Outbreak-associated status is determined at state level in accordance with the respective outbreak case definition for each state. Therefore, HAV cases not classified as outbreak-associated are probably not captured in CDC data. CDC, Centers for Disease Control and Prevention; HAV, hepatitis A virus.

After an intermittent decrease in case rates, a second main peak was observed during May‒December 2020. This peak was driven by case numbers in South Carolina, Kansas, Texas and Georgia. The number of states that had donors affected increased, in parallel with the incidence rates, to 30 by December 2020 (Figure 2). The plasma donor case number map for December 2020 matches the CDC case number map (Figure 3) (*7*). One exception to this pattern is the state of Texas, where case numbers among plasma donors have lately increased, but so far no outbreak-associated cases were reported. Overall, data suggest that HAV outbreaks in the United States have been spilling over into the plasma donor population.

### HAV Biomarker Results for Donors who Showed Seroconversion

To assess the course of infection and immune response in the context of asymptomatic or subclinical HAV infection, we analyzed donations from 10 donors who had positive results (>1 HAV RNA positive donation based on routine testing) by using a panel of nonroutine analytics. The panel included quantitative HAV RNA and ALT assays, semiquantitative HAV IgM and IgG assays, and HAV genotyping.

**Figure 4:**
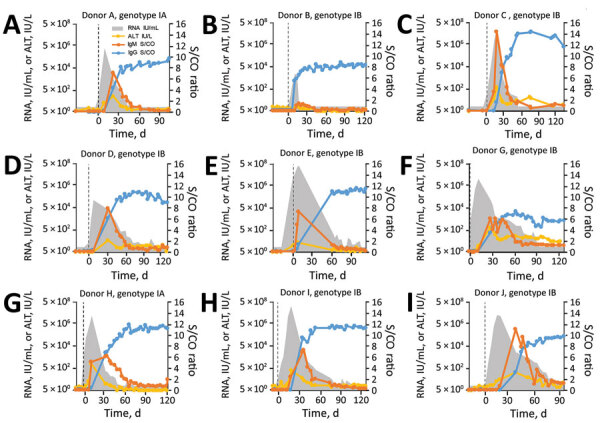
HAV biologic and clinical marker dynamics in plasma donors, United States. Quantitative HAV RNA and ALT results, as well as semiquantitative HAV IgM and IgG results, are shown for all 9 donors who seroconverted. Vertical dashed lines indicate day 0, the earliest collection date with detectable HAV RNA. Reactive nucleic acid testing results below the validated limit of detection of 14 IU/mL are not shown. The IgG result is defined as nonreactive/reactive for S/CO ratios <1 and ≥1, respectively. The IgM assay is defined as nonreactive/reactive for S/CO ratios <0.80 and >1.20, respectively, and as gray zone reactive for S/CO ratios of 0.80‒1.20. The results of a tenth donor (donor F) who had 1 confirmed weak-positive HAV RNA result and all nonreactive IgM/IgG results is not shown but is described in the Results. The figure illustrates the extensive individual variation in viremia curves and timing of biomarker events. ALT, alanine aminotransferase; HAV, hepatitis A virus; S/CO, signal-to-cutoff ratio.

For 9 of 10 donors, HAV infection was clearly confirmed; these donors eventually showed seroconversion for HAV IgM and IgG, and 8 of them showed a transient increase in ALT (Figure 4). The finding that 2 (22%) of these donors were infected with HAV genotype IA and 7 (78%) with IB matches the genotype distribution reported for the current outbreak (15% IA, 84% IB, and <1% IIIA [*15*]). The overall sequence of events for HAV-infected plasma donors was similar to that reported for symptomatic patients: the HAV RNA peak typically preceded the ALT and IgM peak, which in turn preceded the IgG plateau, albeit with considerable individual variation in amplitude and timing (compare donors B, C, and J). Donor B had a particularly interesting profile: low RNAemia, weak IgM response, no increase in ALT, and an unusually early IgG response.

RNA curves obtained showed a maximum HAV RNA titer of 3.1 × 10^8^ IU/mL whereby peak titers >10^7^ IU/mL were common (6/10 donors). Earlier studies had reported an even higher RNAemia of 8.59 × 10^8^ IU/mL (*16*), but suggested lower typical peak titers (*16*,*17*). Although the early phase of infection was characterized by rapid exponential growth and a median estimated HAV doubling time of 17.5 (range 14.1–24.7) hours (Table), the later phase often showed a more or less pronounced biphasic decrease, resulting in a skewed or shouldered RNA curve. Duration of RNAemia was highly variable among donors (Table) (mean 95 days, median 106 days). This duration was similar to that reported for humans who had symptomatic HAV infection (mean 95 days, range 36–391 days) (*18*) but considerably longer than typically examples of RNAemia curves (*2*).

**Table:** Descriptive statistics of HAV infection and immune response in plasma donors, United States*

Parameter	No. samples	Mean	SD	Median	Range
Time to peak RNA, d†	9	12.2	3.8	11.0	8–19
Peak RNA titer, IU/mL	9	7.77 × 10^7^	1.12 × 10^8^	2.61 × 10^7^	1.2 × 10^4^‒3.1 × 10^8^
Duration of viremia ≥100 IU/mL, d	9	54.8	23.4	55.0	14–88
Duration of detectable viremia, d	9	95.0	33.2	106.0	32–128
HAV doubling time, hours	8	18.0	3.5	17.5	14.1–24.7
Time to first positive IgM result, d†	9	21.1	10.3	19.0	7–37
Time to peak IgM S/CO ratio, d†	9	26.4	10.2	29.0	9–38
Peak IgM signal, S/CO	9	7.6	3.6	7.3	1.4‒14.4
Duration of consecutive positive IgM, d	9	36.1	19.6	42.0	1–59
Days between first and last positive IgM result	9	61.3	37.6	58.0	7–112
Time to first positive IgG result, d†	9	25.2	10.7	29.0	9–37
Plateau IgG signal, S/CO	9	10.0	2.2	10.4	5.7–13.5
Time to peak ALT, d†	8	22.0	10.5	21.0	7–37
Maximum fold-change relative to baseline ALT	8	65.1	65.4	27.0	8–159
Peak ALT titer, IU/L	8	452.1	443.4	270.5	40–1,262
No. donations analyzed/donor	9	28.6	6.4	28.0	19–41

*Parameters listed were extracted based on donors who seroconverted (n = 9); donor F was excluded because of absence of seroconversion. For 4 cases, 1 additional donor was excluded from the analysis: donor B for ALT-related parameters (no visible ALT peak) and donor E for extrapolation of HAV doubling time (no early HAV RNA-positive sample available). ALT, alanine aminotransferase; HAV, hepatitis A virus; S/CO, signal-to-cutoff ratio. †Relative to day 0 (defined as collection date with first detectable HAV RNA).

The first HAV IgM-reactive plasma donations occurred a mean (±SD) of 21 (±10) days from day 0; the first IgG-reactive donations were 25 (± 11) days from day 0. Day 0 is the first collection date that showed detectable HAV RNA. No clear association between key serologic and other parameters could be identified (e.g., higher HAV RNA titers were not associated with a faster IgM or IgG response). Analysis of IgM positivity patterns suggests that IgM remained reliably detectable for a median duration of 42 days (range 1–59 days) (Table) when measured by using the Abbot Architect HAVAb IgM assay. After this period, IgM might remain detectable (<112 days after the first IgM-positive result), but not reliably, as suggested by IgM results fluctuating between positive, equivocal, and negative.

To assess whether liver function was affected during the course of asymptomatic/mild HAV infection, we measured ALT levels in all samples from donors who showed seroconversion. Only donor B lacked an ALT peak (Figure 4). For the other 8 donors, we found a median 27-fold increase in ALT over baseline (range 8- to 159-fold) and increased peak ALT titers (mean 452 IU/L, median 271 IU/L, range 40–1,262 IU/L) (Table). These data suggest that liver function is noticeably impacted during most infections, even if peak ALT levels in plasma donors were typically lower than those reported for cases of acute viral hepatitis in general (300–3,000 IU/L) (*19*) and acute hepatitis A in particular (mean 2,000 IU/L and levels >5,000 IU/L for 10% of cases) (*20*). In several instances, ALT levels remained high for prolonged periods (donors G and J) or showed a second peak (donor C).

### HAV Biomarker Results for Donor F

One of 10 selected donors (donor F) had 1 HAV RNA-reactive donation on the basis of the Cobas DPX Assay but never showed seroconversion. All 13 tested donations from day –10 and day +40 were IgM/IgG negative, and efforts to determine the HAV genotype were not successful. We performed several follow-up analyses to assess whether the initial reactive NAT result (cycle threshold value 35.7) might have been a false-positive result. However, we found no such evidence. First, a second independent HAV RNA test confirmed the low-level reactive result for the donation in question. Second, analysis of 12 immune status markers for common pathogens showed matching results between the index sample and 2 other samples from the same donor. Therefore, a sample or donation mixup is highly unlikely. Finally, analysis of routine donation screening results of 54 HAV RNA-positive donors showed that at least 7 donors (13%) had a positivity pattern similar to that for donor F, who had a single, weak, positive donation among many negative donations. Further analyses are needed to clarify what is occurring in these examples.

## Discussion

Our finding that HAV incidence rates among US-source plasma donors have increased in parallel with those in the general US population is not a concern for patients receiving plasma-derived medicinal products. A final product safety margin is considered adequate if the validated process virus reduction factor clearly exceeds the potential viral load of the starting material (*21*). When an epidemiologic situation worsens, safety is assured through appropriate measures associated with the classical virus safety pillars (e.g., use of NAT to exclude viremic donations [*22*] or an adequate validated process virus reduction capacity).

According to industry standards (*22*), source plasma donations and manufacturing pools are tested for HAV RNA and are excluded from further manufacture if positive. Another common practice is to discard donations with negative test results before or after the donation that tested positive. This procedure is a regulatory requirement in the European Union and ensures that any residual virus titer of the starting material, the plasma pool, is minimal and well controlled. A worst-case residual virus titer (such as defined by the validated LOD of the pool testing assay) is generally taken into account when assessing final product safety.

Whether the incidence rates for source plasma donors can be extrapolated to other blood component donors is still unclear. In any case, such comparisons would be difficult because of probable differences in geographic sourcing, testing algorithms, donation frequencies, and potential differences in vaccination rates.

At least in the early phase of the HAV outbreaks, ≈57% of cases reported to CDC were associated with risk factors such as drug use or homelessness (*10*). Although our study did not include a retrospective risk factor investigation, which is a limitation, we consider it unlikely that these risk groups would be greatly represented in the donor population. Donors undergo a careful selection process, which includes extensive measures to prevent plasma collection from risk groups (e.g., medical examination, questionnaire addressing drug use, risk-based drug testing, proof of postal address). Although the donor selection process cannot fully exclude rare cases of noncompliance, it is only 1 of 3 complementary safety pillars (donor screening, donation testing, validated pathogen clearance) that have tremendously improved the safety of plasma products over the past few decades. A potential alternative explanation is that the outbreaks have spread from risk groups to the broader population, including plasma donors. A similar spillover was recently reported for an HAV outbreak that started among men who have sex with men (*23*). Perhaps a narrow confinement within risk groups, even if these are the drivers of an outbreak, cannot necessarily be expected for a highly resistant virus that often causes asymptomatic infections and undergoes fecal‒oral transmission (*1*).

One aspect we observed is a shift in local HAV hotspots over time. Although collection centers in Indiana, Ohio, Tennessee, Pennsylvania, and Florida accounted for 57% of cases observed during 2018 (n = 65) and 59% of all cases observed during 2019 (n = 164), they represented only 13% of all cases during 2020 (n = 92). This abrupt decrease might indicate that measures taken by public health officials, such as information campaigns and vaccination programs, are beginning to bear fruit. Nevertheless, a confounding influence of coronavirus disease‒related measures (e.g., heightened hygiene and social distancing) cannot be entirely excluded.

Our finding that most infected donors showed increased HAV RNA peak titers confirms that persons who have an asymptomatic/subclinical HAV infection might potentially be highly infectious. For instance, in a transfusion setting, as long as neither donor nor recipient have neutralizing antibodies, plasma RNA levels are typically expected to correlate with virus transmission risk. Therefore, adequate measures to prevent HAV transmission are needed.

Whether the protracted low-level RNAemia observed during the late phase of infection is relevant from a pathophysiologic perspective is less clear and might depend on the HAV variant circulating. Although naked HAV virions are expected to be neutralized by HAV antibodies, this assumption might not apply to the more recently described quasi-enveloped HAV particles (*3*) and capsid-free HAV genomes, which are infectious because the RNA plus-strand orientation enables direct use as messenger RNA (*24**,**25*). The host cell entry of both of these variants is mediated by exosomes and relies on distinct host cell factors not used in the same way by naked virions (*25*).

On the basis of the unusual biomarker pattern (Figure 4), it is conceivable that donor B has had previous exposure to an HAV vaccine or natural HAV. Donor B had no ALT response, the lowest peak RNA (12,376 IU/mL), the lowest virus doubling time (24.7 hours), a weak IgM response, and a fast IgG response (positive IgG within 11 days after the first HAV RNA-positive result).

Clinical studies indicate that a small percentage of HAV vaccinees do not reach protective antibody titers or seroconversion after a single dose (*26*,*27*). Similarly, although natural HAV infection is generally believed to induce lifelong immunity (*1*,*9*), exposure to low infectious doses might not always result in a detectable humoral response. This finding highlights the case of donor F, who, after a single confirmed HAV RNA-positive donation, did not seroconvert and declared to never have been vaccinated against HAV. Finally, in rare instances, vaccinated or naturally infected persons might lose their IgG and become susceptible again. For example, a transient, asymptomatic HAV reinfection was reported for 1 patient (albeit one with detectable previous HAV immunity) who had received contaminated erythrocytes (*13*).

Virus doubling time is a useful infection parameter that has potential implications for virus transmission risk. Doubling time is probably influenced by both virus- and host-specific parameters, such as the initial number of infected cells, virus replication and egress strategy, or host cell metabolism. The following doubling times have been reported for bloodborne viruses in humans: HBV, 62.4 hours (range 31 hours−15 days) (*28*); HIV, 15.6 hours (range 8.9 hours–62.6 hours) (*29*); and HCV, 10.8 hours or 17.8 hours (range not specified) (*30*,*31*). HAV doubling time (median 17.5 hours, range 14.1–24.7 hours) partially overlaps with that reported for HCV. Whether this finding is the result of similarities (*32*) such as being a hepatotrophic plus-strand RNA virus remains unknown.

In summary, in parallel with HAV incidence rates in the general population in the United States, HAV infections among US source plasma donors have increased several-fold since January 2016. We leveraged the donors’ frequent donation pattern to capture full RNA, ALT, and HAV IgM/IgG curves. This highly granular biomarker data consolidates our understanding of HAV infection and represents a highly useful resource for clinicians and other public health stakeholders.
